# Analysis of *IFT74 *as a candidate gene for chromosome 9p-linked ALS-FTD

**DOI:** 10.1186/1471-2377-6-44

**Published:** 2006-12-13

**Authors:** Parastoo Momeni, Jennifer Schymick, Shushant Jain, Mark R Cookson, Nigel J Cairns, Elisa Greggio, Matthew J Greenway, Stephen Berger, Stuart Pickering-Brown, Adriano Chiò, Hon Chung Fung, David M Holtzman, Edward D Huey, Eric M Wassermann, Jennifer Adamson, Michael L Hutton, Ekaterina Rogaeva, Peter St George-Hyslop, Jeffrey D Rothstein, Orla Hardiman, Jordan Grafman, Andrew Singleton, John Hardy, Bryan J Traynor

**Affiliations:** 1 Laboratory of Neurogenetics, National Institute of Aging, NIH, Bethesda, MD, USA; 2 Department of Neurology, Washington University School of Medicine, St. Louis, Missouri, USA; 3 Department of Pathology & Immunology, Washington University School of Medicine, St. Louis, Missouri, USA; 4 Department of Clinical Neurological Sciences Royal College of Surgeons in Ireland, Dublin, Ireland; 5 Centre for Clinical Neurosciences, University of Manchester, Greater Manchester Neurosciences Centre, Hope Hospital, Salford, UK; 6 Department of Neuroscience, Via Cherasco 15, 10126 Turin, Italy; 7 Cognitive Neuroscience Section, National Institute of Neurological Diseases and Stroke, Bethesda, MD 20892, USA; 8 Department of Neuroscience, Mayo Clinic College of Medicine, 4500 San Pablo Road, Jacksonville, Florida, USA; 9 Department of Medicine and Physiology, Center for Research in Neurodegenerative Diseases, University of Toronto, Toronto, Canada; 10 Department of Neurology and Neuroscience, Johns Hopkins University, Baltimore, Maryland, USA; 11 Department of Neurology, Beaumont Hospital, Dublin 9, Ireland; 12 SDGE, National Institute of Mental Health, Bethesda, Maryland, USA

## Abstract

**Background:**

A new locus for amyotrophic lateral sclerosis – frontotemporal dementia (ALS-FTD) has recently been ascribed to chromosome 9p.

**Methods:**

We identified chromosome 9p segregating haplotypes within two families with ALS-FTD (F476 and F2) and undertook mutational screening of candidate genes within this locus.

**Results:**

Candidate gene sequencing at this locus revealed the presence of a disease segregating stop mutation (Q342X) in the intraflagellar transport 74 (*IFT74) *gene in family 476 (F476), but no mutation was detected within *IFT74 *in family 2 (F2). While neither family was sufficiently informative to definitively implicate or exclude *IFT74 *mutations as a cause of chromosome 9-linked ALS-FTD, the nature of the mutation observed within F476 (predicted to truncate the protein by 258 amino acids) led us to sequence the open reading frame of this gene in a large number of ALS and FTD cases (n = 420). An additional sequence variant (G58D) was found in a case of sporadic semantic dementia. I55L sequence variants were found in three other unrelated affected individuals, but this was also found in a single individual among 800 Human Diversity Gene Panel samples.

**Conclusion:**

Confirmation of the pathogenicity of *IFT74 *sequence variants will require screening of other chromosome 9p-linked families.

## Background

Amyotrophic lateral sclerosis (ALS, Online Mendelian Inheritance in Man (OMIM) 105400) is characterized by progressive motor neuron degeneration resulting in paralysis and death, usually from respiratory failure, within 3 to 5 years of symptom onset[1]. ALS is typically sporadic in nature. However, 5–10% of cases are familial, and the identification of causal mutations has provided insight into the disease processes that lead to neurodegeneration [2-5]. Frontotemporal dementia (FTD, OMIM 600274) is a degenerative disorder of the frontal and anterior temporal lobes [6] and is the second most common cause of dementia accounting for approximately 20% of pre-senile cases [7]. The syndrome is characterized clinically by initial behavioral and psychological disturbances, followed by cognitive decline eventually leading to dementia and death within a median of seven years from symptom onset [8]. There is a family history of dementia in over 40% of FTD cases suggesting genetic components [8].

Clinical and pathological data indicate that ALS and FTD can form a spectrum of disease [9]. Approximately 5% of ALS patients have clinically florid dementia (ALS-FTD) [10] and roughly half of patients with "classical" ALS have subtle frontal and temporal lobe impairment [11]. Many sporadic and familial FTD cases similarly develop clinical symptoms of motor neuron involvement during the course of their illness [12,8]. Furthermore, ubiquitin inclusions and dystrophic neurites are the hallmark neuropathological findings common to ALS without cognitive impairment, ALS with cognitive impairment, ALS-FTD and "pure" FTD with a greater distribution and load of lesions being associated with cognitive impairment [13].

**Figure 1:**
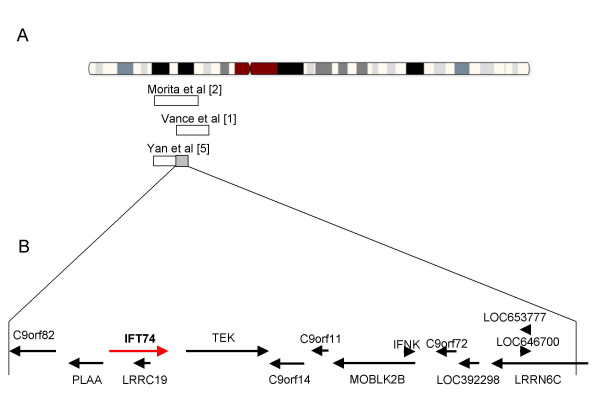
Ideogram of the Chromosome 9p ALS-FTD locus. (A) ideogram of chromosome 9 showing the linked areas defined by Morita et al, Vance et al and Yan et al in 2006. The shared region flanked by D9S1678 and D9S2154 is shaded in grey. (B) Schematic representation of the known genes and predicted transcripts in the area shared by the Dutch, Scandinavian and North American families. In red, IFT74 where sequence variants associated with ALS-FTD, ALS and FTD were identified.

Recently, linkage of ALS-FTD in Dutch and Scandinavian families with apparently autosomal dominant disease was ascribed to a 9.8 megabase (Mb) region at chromosome 9p13.2–21.3 [14,15]. This linkage was replicated and additional haplotype information from seven American ALS-FTD kindreds narrowed the 9p locus to a 2.1 Mb region flanked by D9S1678 and D9S2154 containing 14 genes (figure 1) [16]. We identified two families from the United States that were potentially linked to the 9p locus. Using these families, we undertook a methodical assessment of candidate genes in an attempt to identify the underlying genetic lesion responsible for disease.

## Methods

### Subjects and samples

**Figure 2:**
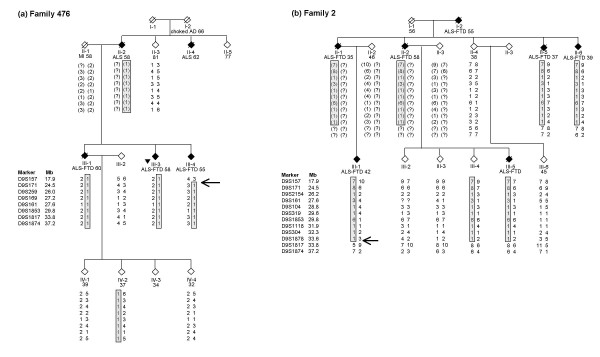
Pedigrees showing affection status, individual IDs, and haplotypes for (a) family 476 and (b) family 2. Gender has been masked to protect the anonymity of the families. Probands are indicated by arrows; open symbol, unaffected; shaded symbol, affected; diagonal line, deceased; current age, or age at death and cause of death, is indicated. AD, Alzheimer Disease. Brackets around an allele indicate an inferred haplotype. Arrows show the limits of the haplotype.

**Figure 3:**
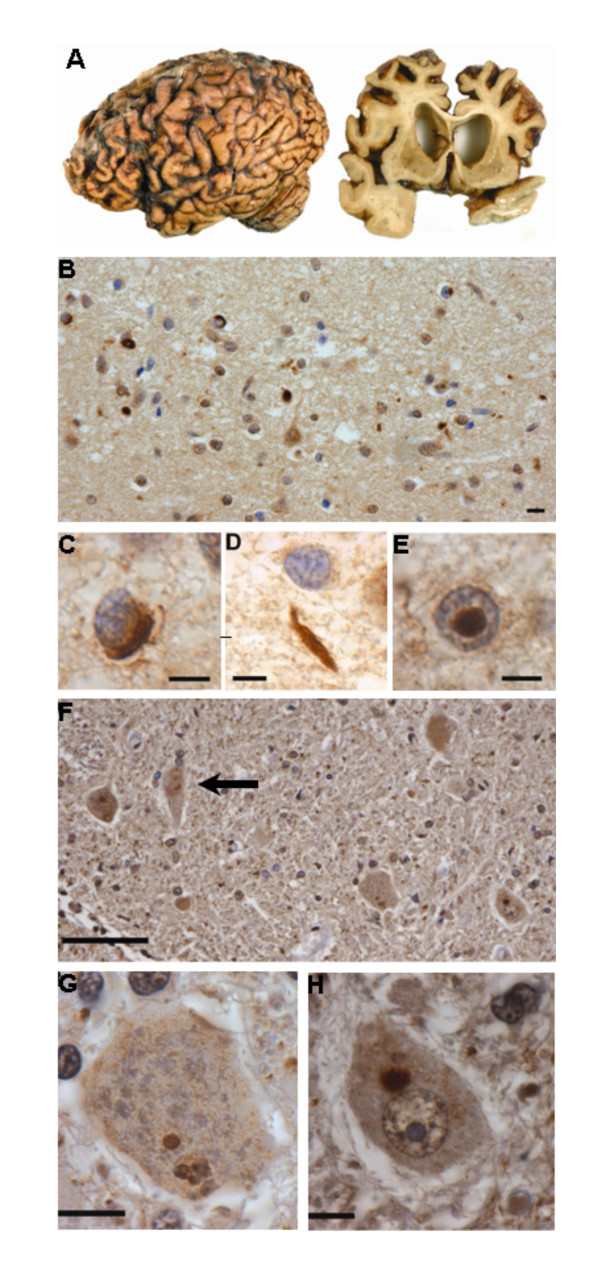
(A) On the left, hemi-brain from individual III-1 of family 476 diagnosed with FTD showing marked frontal and anterior temporal lobe atrophy. On the right, a coronal slice showing severely dilated lateral ventricles with narrowed gyri and widened sulci; (B) ubiquitin-positive, tau-negative inclusions in the superficial laminae of the middle frontal gyrus (arrow, scale bar = 10 nm); (C) high power photomicrograph of a neuronal cytoplasmic inclusion; (D) high power photomicrograph of a dystrophic neurite in the neuropil; (E) high power photomicrograph of a neuronal intranuclear inclusion (C -E, bar = 5 nm); (F) Ubiquitin-positive cytoplasmic inclusions in motor neurons of the lower medulla-spinal cord (arrow, bar = 50 nm); (G) higher power photomicrograph of a neuron containing a cluster of Bunina body-like inclusions; (H) higher power photomicrograph of a loosely aggregated cytoplasmic inclusion (G & H, ubiquitin immunohistocemistry, bar = 5 nm).

F476 was a four-generation, 15-member North American ALS-FTD kindred. The proband (figure 2a, III-3) was a 58-year-old male with a seven year history of behavioral FTD symptoms who subsequently developed ALS during the course of his illness. His younger brother (III-4) developed perseveration, lack of insight and dysregulation of social and interpersonal conduct at the age of 52. He later developed motor weakness and remains alive three years after disease onset. An older brother (III-1) began showing poor judgment and inappropriate behavior at 50 years of age. He died twelve years later with severe dementia. Marked frontal and anterior temporal lobe atrophy was detected at post-mortem. Histology revealed the stereotypical features of frontotemporal lobar degenerations (FTLDs): neuronal loss, microvacuolation, and gliosis (figure 3). The frontal lobe was the most severely affected region with focal trancortical neuronal loss. Ubiquitin immunohistochemistry revealed neuronal cytoplasmic inclusions, sparse neuronal intranuclear inclusions, and dystrophic neurites identical to those seen in familial and sporadic cases of FTLD with ubiquitin-positive, tau-negative inclusions (FTLD-U). There were Bunina bodies in the motor neurons of the anterior horns of the cervical spinal cord, but there was no evidence of corticospinal tract degeneration seen in cases of motor neuron disease. This case had additional Alzheimer's disease-type pathology, namely diffuse beta-amyloid plaques in the absence of neuritic plaques or neurofibrillary tangles. Autopsies and neuropathological procedures were performed according to the protocols of the Washington University Alzheimer's Disease Research Center neuropathology Core.

F2 was a three-generation, 16-member North American kindred, in which seven individuals had been diagnosed with ALS-FTD (figure 2b).

Patients with ALS fulfilled the El Escorial criteria for probable or definite ALS and were diagnosed by a consultant neurologist after exclusion of ALS mimic syndromes [17]. Frontotemporal dementia was diagnosed by clinical and neuropsychological criteria. The diagnosis was confirmed by autopsy in one familial ALS-FTD case.

### Marker analysis

DNA was extracted by standard procedures after ethically approved, written informed consent was obtained. Ethical approval for collection of DNA and clinical phenotype information was provided by the National Institute of Aging Institutional Review Board, Baltimore, MD (protocol #2003-081). Polymerase chain reaction (PCR) was performed to amplify DNA with markers spaced across the known chromosome 9p21 locus. Markers were selected from the Applied Biosystems Prism Linkage Mapping Set Version 2.5 (ABI, Foster City, CA). Additional makers were chosen based on mapping information publicly available from the UNISTS database at the National Center for Biotechnology Information (NCBI). For allele identification, the PCR products were separated and scored using automated ABI 3100 sequencing equipment.

### Sequencing of candidate genes

The 14 genes within the 2.1 Mb region of the 9p locus defined by the Dutch, Scandinavian and North American families[14-16] were identified from the NCBI [18] and Ensembl databases [19]. Genomic primer design for all genes was performed using ExonPrimer [20]. Each exon and at least 30 bp of flanking intronic sequence was PCR amplified using primer pairs (available upon request) using whole genome amplified DNA (Repli-G, Qiagen Inc., Valencia, CA) from affected individuals of each family used. PCR amplification was carried out using Qiagen HotStarTaq master mix polymerase, and 10 pmol of both forward and reverse primers per the manufacturer's instructions (Qiagen Inc.). Each product was sequenced using forward and reverse primers with Applied Biosystems BigDye terminator v3.1 sequencing chemistry as per the manufacturer's directions. The resulting reactions were run on an ABI3100 genetic analyzer and analyzed with Sequencher software (version 4.5, GeneCodes Corp., MI). Detected sequence variants were confirmed by repeat PCR amplification and sequencing using original genomic DNA. In addition to these 14 genes, we also sequenced an additional 47 genes in the larger haplotype before the report [16] limiting the linked haplotype became public (Table 1, primers available upon request). Coding and flanking regions of Cu/Zn superoxide dismutase (*SOD1)*, microtubule associated protein tau (*MAPT) *and progranulin (*GRN) *genes known to be associated with ALS-FTD[21-24], had been sequenced for mutations as previously described[25,22].

### Mutation assay

The presence of *IFT74 *sequence variants were assessed in 500 North American control subjects, 9 ALS-FTD cases with a positive family history of ALS-FTD, 11 cases of ALS-FTD without a family history, 164 sporadic FTD cases, 31 cases of familial FTD, 127 Irish cases of ALS and 83 North American cases of ALS (see Table 2 for list of samples). In addition, we used 800 DNA samples from the Centre d'Etude du Polymorphisme Humain (CEPH) Human Genome Diversity Panel (HGDP), a resource of individuals from 51 different world populations. Information for this panel is limited to sex of the individual and population origin [26]. To identify the Q342X sequence variant, a genomic fragment was amplified using the primer pair 5'-TGGAAAAGATATCTAACCTCCCC-3' and 5'-CCCAGGTAGTTGAACAGTCTCTG-3'. The resulting 262 bp fragment was sequenced as described above. The remaining 18 exons of *IFT74 *were also amplified and sequenced in the patient samples to determine the presence of sequence variants in the rest of the gene (see table 3 for list of primers).

### cDNA amplification

cDNA from a marathon Rapid Amplification of cDNA Ends (RACE)-ready adult human brain library (BD Biosciences, CA) was used as a template to amplify overlapping fragments of the predicted 2 kilobase (kb) transcript. Overlapping primers within each exon were designed using ExonPrimer, and PCR and sequencing were carried out as described above.

### Western blotting

Human brain soluble extracts (40 ug per lane) were separated on 4–20% sodium dodecyl sulphate – polyacrylamide gel electrophoresis (SDS-PAGE) gels and blotted using a goat polyclonal antibody to the C-terminus of IFT74 (anti-CMG1, Everest Biotech Ltd, Oxfordshire, UK) at a final antibody concentration of 0.5 ug mL^-1^. For competition experiments, antibody was pre-incubated with a 10-fold excess (w/w) of immunizing peptide (KTIVDALHSTSGN).

### Confocal microscopy

Primary cortical neurons were prepared from E18 rat pups using papain dissociation and were plated on poly-L-lysine coated glass coverslips at 10^6 ^cells per dish. After 5 days *in vitro*, cells were fixed with 4% paraformaldehyde, permeabilized with 0.1% saponin and stained with the goat polyclonal antibody to IFT74 at a concentration of 2.5 mg mL^-1^. Secondary antibody was donkey anti-goat IgG conjugated to AlexaFluor 488 (1:200, Molecular Probes, Carlsbad, CA) and nuclei were identified with TO-PRO3 (Molecular probes). Coverslips were imaged with a Zeiss LSM510 META confocal microscope using consistent gain and offset settings for samples stained with antibody alone or with antibody plus immunizing peptide. Secondary antibody alone gave no signal.

## Results

Previous linkage analysis of F2 using dinucleotide marker data had revealed a single region with a lod score of ~1.5 on chromosome 9p that matched with the published chromosome 9p21.3-p13.3 ALS-FTD locus (figure 2b). Similarly, marker data showed a haplotype across chromosome 9p that segregated with disease status in F476 (figure 2a). Based on these data, F476 and F2 were selected for mutational screening of the 14 candidate genes within the previously defined 2.1 Mb region of the chromosome 9p ALS-FTD locus.

**Figure 4:**
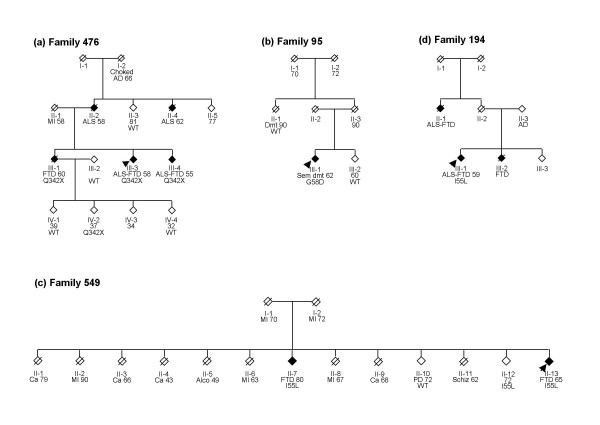
Family 476, 95, 549 and 13 originate from North America. WT indicates a mutation-negative subject, Q342X, G58D and I55L indicate subjects carrying a heterozygous sequence variation. Currently unaffected mutation carriers have not been shown; in all instances these individuals have not yet reached the maximum age at onset observed.

**Figure 5:**
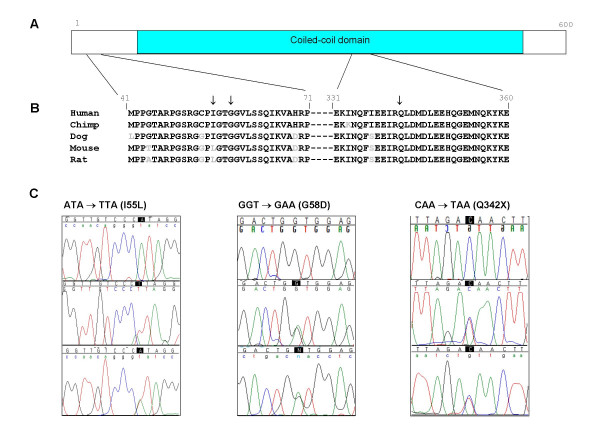
Sequence variants in *IFT74 *are linked to Chromosome 9p ALS-FTD. (A) Schematic of IFT74's structure. (B) Sequence variants I55L, G58D and Q342X are shown relative to affected protein domains and are indicated by black arrowheads. Human IFT74 protein aligned with other orthologs from chimpanzee (XP_520518.1), dog (XP_531964.1), mouse (AAH23760.1) and rat (NP_001007002.1). Amino acid number is based on the human protein. (C) chromatograms showing wild type (top), forward mutant (middle) and reverse mutant (bottom) sequences of I55L (left), G58D (middle) and Q342X (right).

In the course of sequencing the intraflagellar transport gene (*IFT74*), we identified a C to T sequence variant at nucleotide 1024 in exon 13 in the proband of F476 (III-3, figures 4a and 5). This base pair change predicts a premature stop codon at position 342 of the peptide (Q342X) truncating the last 258 residues. This variant segregated with disease within the family as it was present in the two brothers of the proband diagnosed with ALS-FTD (III-1, III-4). The Q342X sequence variant was not present in four unaffected individuals within the kindred (II-3, III-2, IV-2 & IV-4), in 1,000 chromosomes from North American controls or in 900 chromosomes from the Human Genome Diversity Panel although this C to T base pair change is ostensibly in a library clone derived from human thymus (BX436367, clone CS0CAP001YM04, Life Technologies, Inc). One younger unaffected individual carried the Q342X sequence variant. We did not find *IFT74 *sequence variants in F2 by sequencing or exonic gene dosage methods (data not shown). Neither F476 or F2 had additional mutations in the other 13 genes within the 9p locus defined by the Dutch, Scandinavian and North American families[14-16] or in 47 additional genes in the larger haplotype.

In order to assess the prevalence of *IFT74 *sequence variants, we sequenced the entire coding region of this gene in a large number of ALS, ALS-FTD and FTD samples (Table 2). The Q342X mutation was not found in any of these patients. However, we identified a G58D (nt173 G to A) sequence variant in a Caucasian woman who developed sporadic semantic dementia without motor involvement at the age of 58 (II-1, figures 4b and 5). This G58D sequence variant was not found in 900 chromosomes from North American controls or in 1,600 chromosomes from the HGDP. We also identified an I55L sequence variant (nt163 A to T) in three additional affected probands; first, the I55L sequence variant was found in the proband of F549 who had been diagnosed with FTD at the age of 62 (figures 4c and 5). His 80-year-old brother (II-7) had been diagnosed with FTD at the age of 75 and carried the I55L sequence variant. A 70-year-old apparently unaffected sister (II-12) also carried the I55L sequence variant; second, the proband of F194, a 59-year-old Caucasian woman diagnosed with ALS-FTD, had the I55L sequence variant. Her sister had died of FTD and her paternal uncle had died of ALS-FTD (DNA samples not available); third, the I55L sequence variant was found in a 67-year-old man with sporadic ALS. He had presented one year before his death with bulbar symptoms including emotional lability, and had become increasingly withdrawn and indifferent to his symptoms during the course of his illness. I55L was not found in 900 chromosomes from North American controls, but it was found in 1 of 1,600 chromosomes from the HGDP in a French sample.

**Figure 6:**
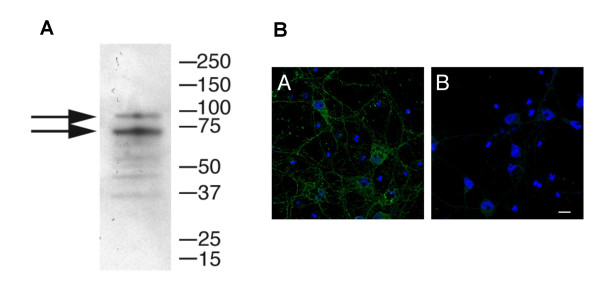
(A) Human brain soluble extracts (40 ug per lane) were separated on 4–20% SDS-PAGE gels and blotted using a goat polyclonal antibody to the C-terminus of IFT74 (Imgenex, anti-CMG1) at a final antibody concentration of 0.5 mg mL^-1^. Two major bands at ~90 kDa and ~70 kDa were identified which were blocked by pre-absorption of the antibody with the immunizing peptide (data not shown), indicating specificity. (B) A. Immunofluorescent staining of primary rat cortical neurons with the same antibody showed localization of IFT74 to vesicles in the cell body and along the neuronal processes. B. Secondary antibody alone gave no signal using consistent gain and offset settings.

We proceeded to verify that IFT74 is expressed in brain. Refseq (NCBI) provisionally reports the *IFT74 *gene to contain 14 coding exons based on NM_025103. We sequenced human brain derived cDNA and found a T to A transversion at nt599 of NM_025103 (primers available upon request). This base pair substitution converts a stop codon at position 159 to lysine indicating that *IFT74 *contains 19 exons encoding a 600 residue protein with a predicted molecular weight of 69.2 kDa. This larger coding region of *IFT74 *was confirmed by deposited sequences AY040325 and CR617782, which show lysine at codon position 159. Western blotting of human brain lysates with a polyclonal antibody against a peptide from the C-terminus of IFT74 demonstrated two major bands at ~90 kDa and ~70 kDa, of which the lower band is the most prominent (figure 6a). Both of these bands were blocked by pre-absorption of the antibody with the immunizing peptide (data not shown), indicating specificity. These data show that this gene is expressed throughout the adult human brain and also confirmed that *IFT74 *contains 19 exons. Staining of primary rat cortical neurons with the same antibody demonstrated that IFT74 is localized to vesicles in the cell body and along the neuronal processes (figure 6b).

## Discussion

We found sequence variations in the *IFT74 *gene in patients with ALS-FTD, FTD and sporadic ALS. Five pieces of genetic information suggest that the *IFT74 *sequence variants are relevant to disease pathogenesis. First, the nonsense sequence variant (Q342X) segregates with disease in a small ALS-FTD kindred. Second, this premature stop codon significantly truncates the IFT74 protein in a manner likely to have a critical effect on the function of this plausible candidate gene. Third, additional sequence variants (G58D and I55L) were found in the *IFT74 *gene in other disease cases. Fourth, we have sequenced every known gene and predicted transcripts in the candidate region as defined by the minimal interfamily haplotype shared by the Dutch, Scandinavian and North American ALS-FTD families (figure 1) and the gene encoding *IFT74 *was the only gene that contained variants not identified in the general population and fifth, these mutations are not present in 900 to 1,000 North American control chromosomes.

Against these five pieces of suggestive information are four pieces of evidence against pathogenicity. First, we failed to find a mutation in the second family we used in our screening (F2). Second, the fact that the stop mutation is in the cDNA database in a sample of unknown provenance, third the fact that the I55L mutation is in a sample from France in the CEPH diversity series argues against the pathogenicity of that particular mutation. The genetic linkage reports indicate that mutations at the chromosome 9p locus are incompletely penetrant [14,15] and this complicates the interpretation of both segregation data within families and of variants in control populations. Fourth: on contacting the senior authors of the original linkage report [14], they were unable to find a point variant in their linked family.

## Conclusion

Our data indicate that IFT74 is a 600-residue, 69-kDa coiled-coil containing protein that localizes to the intracellular vesicle compartment. This protein is a component of the intraflagellar transport system responsible for vesicular transport of material synthesized within the cell body into and along the dendritic and axonal processes of human neurons[27]. The importance of vesicle synthesis and axonal transport in motor neuron disease is increasingly recognized. Vesicle associated protein B missense (VAPB) mutations have been identified in familial ALS [5] and the wobbler mouse, an animal model of ALS, is caused by mutations in the vesicular protein sorting factor 54 [28]. Dynactin is the motor protein responsible for retrograde axonal transport and mutations in the p150 subunit of this complex have been described in patients with ALS [29] and ALS-FTD [30]. Furthermore, a mutation in the antegrade axonal transport kinesin gene, KIF1B, has been described in a single family with Charcot-Marie-Tooth disease-2A1, a hereditary motor and sensory axonal neuropathy (OMIM 118210). Given its role in vesicle transport, IFT74 is a plausible biological candidate to explain the neurodegeneration characteristic of both ALS and FTD phenotypes in a subset of patients. However, as the Q342X mutation was found in a cDNA library and in an (as yet) unaffected family member, there is currently no clear evidence that this mutation is of causal significance and *IFT74 *may just be a risk factor for ALS-FTD. Clearly, more work will be needed to determine its role, if any.

### Accession numbers

Intraflagellar transport 74 homolog (*IFT74*, also known as capillary morphogenesis protein 1 (CMG1); coiled-coil domain containing 2 (CCDC2); FLJ22621): GeneID 80173; UniGene Hs.145402; mRNA: AY040325 (gi:15418996); protein: AAK77221 (GI:15418997); *SOD1 *(NM_000454); *MAPT *(NM_016835); *GRN *(NM_002087).

### Online Mendelian inheritance in man [31]

Amyotrophic lateral sclerosis [ALS, OMIM 105400]; Fronto-temporal dementia [FTD, OMIM 600274]; Fronto-temporal dementia/amyotrophic lateral sclerosis [ALS/FTD, OMIM 105550], Intraflagellar transport protein 74 (*IFT74*), previously CCDC2, CMG1 [OMIM 608040]

## Abbreviations

ALS, amyotrophic lateral sclerosis; FTD, frontotemporal dementia, IFT74, intraflagellar transport 74 homologue; OMIM, online Mendelian inheritance in man; FTLD, frontotemporal lobar degeneration; FTLD-U, FTLD with ubiquitin-positive, tau-negative inclusions; MND, motor neuron disease; NCBI, national center for biotechnology information; SOD1, superoxide dismutase one; MAPT, microtubule-associated protein tau; GRN, granulin; CEPH, Centre d'Etude du Polymorphisme Humain; HGDP, Human Genome Diversity Panel; cDNA, complementary DNA; RACE, Rapid Amplification of cDNA Ends; SDS-PAGE, sodium dodecyl sulfate-polyacrylamide gel electrophoresis; CMG1, capillary morphogenesis protein 1; Refseq, reference sequence; KIF1B, kinesin family member 1B.

## Competing interests

The author(s) declare that they have no competing interests.

## Authors' contributions

PM, JS and SJ contributed equally to this work. PM, JS, SJ and SB carried out the molecular genetic studies, participated in the sequence alignment and drafted the manuscript. AS, JH and BJT conceived of the study, participated in its design and coordination, performed data analysis and drafted the manuscript. MRC and EG carried out the immunoassays and drafted the manuscript. NJC and DMH performed the autopsy, provided the photomicrographs and participated in writing the manuscript. MJG, SP, AC, HCF, EH, EW, JA, MLH, ER, PS, JDR, OH and JG were involved in ascertaining patients, providing phenotype data, obtained DNA samples and participated in editing the manuscript. All authors read and approved the final manuscript.

## Pre-publication history

The pre-publication history for this paper can be accessed here:



## Figures and Tables

**Table 1:** Genes in the extended Chromosome 9p ALS-FTD locus* that were excluded as candidate genes by sequencing

***Start bp***	***Symbol***	***Start bp***	***Symbol***
26,056,673	*LOC441390*	33,245,026	*BAG1*
29,814,856	*LOC286239*	33,255,000	*C9orf83*
30,378,933	*LOC401497*	33,278,862	*LOC441393*
30,678,956	*LOC441391*	33,280,510	*NFX1*
30,679,911	*LOC442405*	33,374,948	*AQP7*
30,789,463	*LOC442406*	33,431,162	*AQP3*
30,811,997	*LOC441392*	33,451,352	*NOL6*
31,243,899	*LOC138412*	33,493,783	*LOC441394*
32,323,455	*LOC392301*	33,514,488	*ANKRD18B*
32,374,650	*ACO1*	33,570,693	*LOC441395*
32,445,705	*DDX58*	33,607,843	*TRBV20OR9-2*
32,530,543	*TOPORS*	33,614,223	*ANXA2P2*
32,543,523	*NDUFB6*	33,619,141	*TRBV21OR9-2*
32,619,452	*TAF1L*	33,623,851	*TRBV22OR9-2*
32,633,108	*LOC389710*	33,628,035	*TRBV23OR9-2*
32,773,497	*LOC401498*	33,639,129	*TRBV24OR9-2*
32,935,943	*LOC392302*	33,652,201	*TRBV25OR9-2*
32,962,608	*APTX*	33,665,438	*PTENP1*
33,009,952	*LOC401499*	33,672,268	*TRBVAOR9-2*
33,015,309	*DNAJA1*	33,685,578	*TRBV26OR9-2*
33,031,762	*LOC401500*	33,776,219	*TRBV29OR9-2*
33,037,169	*SMU1*	33,785,559	*PRSS3*
33,100,642	*B4GALT1*	33,807,565	*UBE2R2*
33,230,196	*SPINK4*		

*as defined by the Dutch and Scandinavian families [14,15]

**Table 2:** Samples in which the *IFT74 *gene was sequenced

	***Whole gene***	***Exon 2***	***Exon 12***
NINDS ALS-FTD and FTD samples [32]	32		
Toronto ALS-FTD and FTD samples [33]	95		
Irish ALS-FTD and ALS samples [34]	127		
Johns Hopkins ALS samples	51		
New York Brain Bank ALS, ALS-FTD and FTD samples	24		
University of Miami/National Parkinson Foundation ALS samples	7		
Miscellaneous ALS, ALS-FTD and FTD samples	84		
North American control samples*		450	500
Human Genome Diversity panel [26]		800	450
**Total**	**420**	**1,250**	**950**

*available from the NINDS Neurogenetics Repository at the Coriell Institute for Medical Research, Camden, NJ

**Table 3:** Primers used to sequence the 19 exons of *IFT74*

	***Sequence***	***Product size***
Exon 1 Forward	TGAACAAAATTAGCCTTGTAGTGG	395
Exon 1 Reverse	AAAAACTGGGGTGCAGTGG	
Exon 2 Forward	CTTTGCAGTTTTACAATAAAAGATGT	400
Exon 2 Reverse	CCATAATTCTGATTACATGCCATA	
Exon 3 Forward	TTGGATTTGCTATTCTGGTGG	172
Exon 3 Reverse	GACAGAAGGTACACAATATTTCGAG	
Exon 4 Forward	AATTTTGCTTAACTAGAGTTTTCTGG	250
Exon 4 Reverse	TGAACATTACAAACAATATGGATAGC	
Exon 5 Forward	TTGTAGCTATCCATATTGTTTGTAATG	282
Exon 5 Reverse	CTTGTTGGCTGCATGTTTG	
Exon 6 Forward	TTTGTTTGTATTTTTGTTTTTAGTTGG	246
Exon 6 Reverse	GCAACTAAGGGAGCTCAAGG	
Exon 7 Forward	TCATTTAGTTCCTAGCCAAAATAACC	230
Exon 7 Reverse	CAAGGAAATAACTGGATCACAAC	
Exon 8 Forward	AGTCACACACTGTGGTTGAATG	343
Exon 8 Reverse	TGCTGATGCTGCTGATATTC	
Exon 9 Forward	TTTCCCAGCTCCCTCCC	304
Exon 9 Reverse	CTAGGATCTTTGTGGGTCCAG	
Exon 10 Forward	CCCCATTCTTATAAACTGAAACC	324
Exon 10 Reverse	AGCTTTACTACTTGCATTAATATCCC	
Exon 11 Forward	TTCAAGAAAGAGAGTAGATTTGACAC	301
Exon 11 Reverse	TGCAATGGCTCTTAAACACTC	
Exon 12 Forward	TGGAAAAGATATCTAACCTCCCC	262
Exon 12 Reverse	CCCAGGTAGTTGAACAGTCTCTG	
Exon 13 Forward	TGACTAATGGCATAGAGAGAACC	240
Exon 13 Reverse	TATCTGCCCAAAATGAGGTC	
Exon 14 Forward	GCCTGGCAACAGAGTGAG	357
Exon 14 Reverse	AAGGGAATTGAAAGGAAGCAG	
Exon 15 Forward	CATCCTTTGACAGTGTTTTCC	297
Exon 15 Reverse	CAATGAGGCTTTAAAATAATCAGAG	
Exon 16 Forward	GAAATTGGTTATTGAGGGGATG	539
Exon 16 Reverse	TTGCTTTTCTTTAAAACTTTAAGTCAC	
Exon 17 Forward	CGGGTTTTAGAGTAGTTATAGCCTTG	381
Exon 17 Reverse	TGGCTGTCTTAAAAGTCAGAAAATC	
Exon 18 Forward	TTTTGTCTTATCCTCTTAGAAAGGC	259
Exon 18 Reverse	TAGCCAGGGTGGTCTCAATC	
Exon 19 Forward	TGCTTAATCTGTTAAAATGATGTACTG	383
Exon 19 Reverse	AATTTTAGGTAAACTCCAAAAGTAAGC	
